# Intra-epidemic evolutionary dynamics of a Dengue virus type 1 population reveal mutant spectra that correlate with disease transmission

**DOI:** 10.1038/srep22592

**Published:** 2016-03-04

**Authors:** Hapuarachchige Chanditha Hapuarachchi, Carmen Koo, Relus Kek, Helen Xu, Yee Ling Lai, Lilac Liu, Suet Yheng Kok, Yuan Shi, Raphael Lee Tze Chuen, Kim-Sung Lee, Sebastian Maurer-Stroh, Lee Ching Ng

**Affiliations:** 1 Environmental Health Institute, National Environment Agency, 11, Biopolis Way, #06-05-08, Singapore 138667; 2 School of Life Sciences and Chemical Technology, Ngee Ann Polytechnic, Block 83, #04-00, 535 Clementi Road, Singapore 599489; 3 Bioinformatics Institute (BII), Agency for Science, Technology and Research (A*STAR), 30 Biopolis Street #07-01, Matrix Building, Singapore 138671; 4 School of Biological Sciences (SBS), Nanyang Technological University (NTU), 60 Nanyang Drive, Singapore 637551; 5 National Public Health Laboratory (NPHL), Ministry of Health (MOH), 3 Biopolis Drive, #05-14 to 16, Synapse, Singapore 138623

## Abstract

Dengue virus (DENV) is currently the most prevalent mosquito-borne viral pathogen. DENVs naturally exist as highly heterogeneous populations. Even though the descriptions on DENV diversity are plentiful, only a few studies have narrated the dynamics of intra-epidemic virus diversity at a fine scale. Such accounts are important to decipher the reciprocal relationship between viral evolutionary dynamics and disease transmission that shape dengue epidemiology. In the current study, we present a micro-scale genetic analysis of a monophyletic lineage of DENV-1 genotype III (epidemic lineage) detected from November 2012 to May 2014. The lineage was involved in an unprecedented dengue epidemic in Singapore during 2013–2014. Our findings showed that the epidemic lineage was an ensemble of mutants (variants) originated from an initial mixed viral population. The composition of mutant spectrum was dynamic and positively correlated with case load. The close interaction between viral evolution and transmission intensity indicated that tracking genetic diversity through time is potentially a useful tool to infer DENV transmission dynamics and thereby, to assess the epidemic risk in a disease control perspective. Moreover, such information is salient to understand the viral basis of clinical outcome and immune response variations that is imperative to effective vaccine design.

Dengue, caused by Dengue virus (DENV), is currently the most prevalent mosquito-borne viral disease. It is estimated to affect over 400 million individuals annually and over 50% of world’s population living in Americas, Southeast Asia and the Western Pacific regions is at risk of DENV infection^1^. DENV infection causes a clinical syndrome that is benign in most patients. The disease can be fatal in a small proportion of the infected due to haemorrhage and fluid leakage that lead to systaemic shock and multi-organ failure^2^. There is neither an effective antiviral agent nor a licensed vaccine against dengue in many endemic settings.

DENV is a positive sense single stranded RNA virus of the genus *Flavivirus* which is transmitted to humans by *Aedes aegypti* and *Aedes albopictus*. The virus genome of approximately 11.8 kb encodes for a single polypeptide arranged in three structural (Capsid-preMembrane-Envelope) and seven non-structural (NS) proteins (NS1-NS2A-NS2B-NS3-NS4A-NS4B-NS5). DENV exists as four phylogenetically and antigenically distinct serotypes (DENV1–4). The exposure to a particular serotype elicits type-specific life-long immunity^3^.

RNA viruses, such as DENV, typically display high mutation rates as a result of the error-prone RNA polymerase activity during genome replication^4^. In addition, the genetic recombination further facilitates their opportunity for genetic novelty^5^. This ultimately results in genetically diverse virus populations consisting of multiple genotypes of monophyletic nature. At a micro-scale, however, RNA virus populations often appear to exist as ensembles of mutant spectra that collectively behave as “quasispecies”^6^. The composition of such mutant ensembles is in a dynamic flux as a result of being subjected to competition, immune pressure, selection and genetic variation^7^. Being an RNA virus, DENV is not an exception. Each DENV serotype is composed of highly heterogeneous genotypes that show a characteristic geographical distribution pattern^8^.

Even though a large number of recent reports are available on the genotype and lineage (macro-scale) diversity of DENV from different regions, only a limited number of studies has focussed on the diversity of homotypic virus populations that circulated during epidemics^9^,^10^,^11^. Even among them, reports of intra-epidemic DENV diversity are largely of macro-scale. Fine-scale descriptions on the dynamics of mutant spectra within DENV populations collected serially during epidemics are extremely scarce^12^, mainly because of the limitations in sampling and genome sequencing. Such micro-scale genetic descriptions are important to decipher the evolutionary dynamics of epidemic strains which generally show a reciprocal relationship with viral transmission^13^ and ecological factors, thereby shaping dengue epidemiology. Similar analyses also enable us to fingerprint epidemic viral lineages and assist in tracking virus movement to better guide disease control operations. In order to achieve this, a thorough virus surveillance programme that routinely monitors local DENV populations is an absolute necessity.

Singapore’s virus surveillance programme offers us an opportunity to characterise circulating DENV strains on a routine basis^14^,^15^. Laboratory confirmed dengue sera obtained through an island wide primary healthcare network are used to determine the serotype and genotype composition of local DENV populations on a weekly basis. Serotype data is used to assess the risk of impending epidemics^14^ and genotype data enables monitoring the emergence and spread of novel strains associated with disease clusters^15^. Singapore experienced its worst known dengue epidemic in 2013^16^ that recorded 40,508 confirmed cases by the end of 2014. This unprecedented increase in dengue incidence was associated with a switch from DENV-2 to DENV-1 in early 2013, together with the emergence of a new lineage of DENV-1 genotype III. In the present study, we describe the fine-scale temporal variations of the epidemic lineage of DENV-1 genotype III obtained from different parts of Singapore. Our aim was to provide a better understanding of the evolutionary dynamics of an epidemic lineage of DENV in relation to its heterogeneity, spatiotemporal distribution and overall disease transmission throughout the expansion of an extensive dengue epidemic.

## Results and Discussion

**Figure 1:**
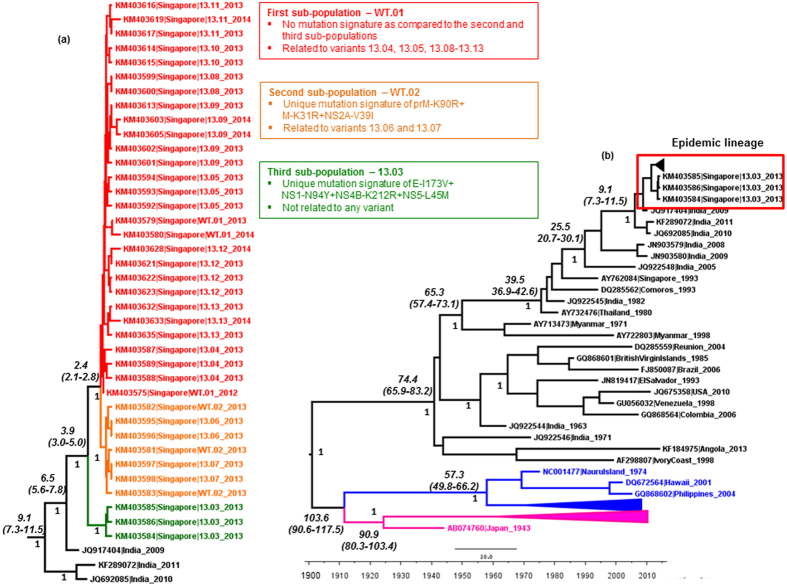
Phylogenetic and tMRCA analysis of the epidemic lineage and DENV-1 genotypes. The maximum clade credibility tree was constructed using the Bayesian Markov Chain Monte Carlo (MCMC) method implemented in the BEAST package v1.7.4^51^,^52^. The analysis included 38 complete polyprotein sequences generated during this study and 44 sequences retrieved from GenBank database. Only a subset of complete poplyprotein sequences representative of each variant was included in the phylogeny to make the tree concise. **(a)**. The analysis of epidemic lineage. The first and second sub-populations of wild-type isolates ancestral to variants 13.04–13.13 are indicated with taxa name WT.01 and WT.02 respectively. The figure demonstrates the genetic relatedness of each variant to the first (red), second (orange) and third (green) sub-populations. **(b)**. Phylogeny and tMRCA analysis of DENV genotypes. Genotypes I, II and III are shown in purple, blue and black respectively. The epidemic lineage is shown in the red box. Numbers shown on branches are posterior probability values (non-italics) of major nodes and tMRCA values in years (italics). The 95% HPD values are shown within brackets. All sequences are named with accession number, country of origin and year of isolation. In addition, the taxon names of study isolates include the variant identity.

A total of 7944 patient sera received with consent were screened at Environmental Health Institute (EHI) Diagnostics during the study period from November 2012 to May 2014. Of them, 2184 (28%) sera were positive for DENV NS1 antigen. DENV serotypes were determined in 2026 (92.8%) NS1-positive sera either by real-time polymerase chain reaction (PCR; n = 1792) or by conventional PCR (n = 234). Of them, 1345 (66.4%) infections were due to DENV-1 of which 1188 virus strains were genotyped based on envelope (*E*) gene sequences. Of total DENV-1 genotyped during the study period, 1058 (89.1%) belonged to a monophyletic lineage of genotype III (Fig. 1). A detailed sample summary is presented in Supplementary Table S1.

DENV-1 genotype III strains of the epidemic lineage were first detected locally in November 2012. The genetic distinction of the epidemic lineage from the locally circulating DENV-1 strains indicated that its emergence in Singapore was likely to be due to an introduction. Based on the available sequences in GenBank, the closest ancestor of the epidemic lineage was an Indian isolate (NCBI: JQ917404) reported in 2009 (Fig. 1). The time to most recent common ancestor (tMRCA) analysis suggested an equivalent time of origin (during 2007–2008) for the epidemic lineage (median 6.5 yrs before 2014; 95% highest posterior density (HPD) 5.6–7.8 yrs) (Fig. 1).

**Figure 2:**
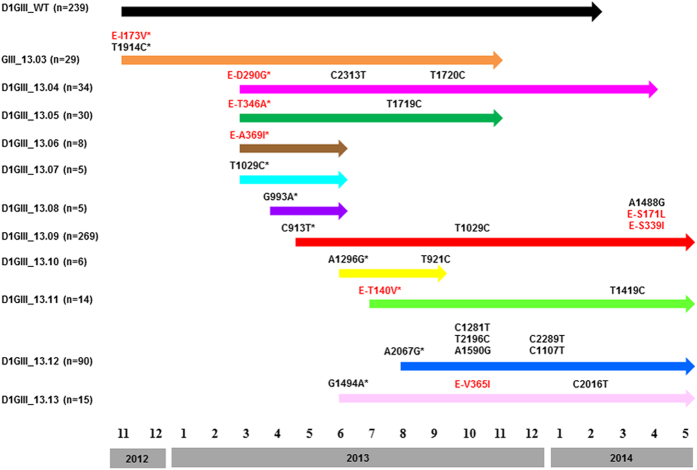
Envelope (*E*) gene mutation map of DENV-1 genotype III epidemic strains/variants. The analysis included 744 complete *E* gene sequences generated from November 2012 to end of May 2014. The figure illustrates temporal pattern of emergence, maintenance and extinction of each variant during the study period. Arrows indicate the transmission period of each variant. A timeline is given below the figure with numbers 1–12 to represent January to December in respective years. Substitutions shown are the *E* gene based signature mutations of each variant. Initial substitutions were named as founder (primary) substitutions and have been shown with an asterisk. Those that appeared later in respective variants were named as non-founder (secondary) substitutions (without an asterisk) in the text. Those shown in red are “fixed” non-synonymous substitutions. Remaining residues are “fixed” synonymous substitutions. Positions of all amino acid substitutions are shown at the *E* gene level, whereas the synonymous substitutions are numbered from the beginning of the structural polyprotein. D1GIII = DENV-1 genotype III, n = number of sequences belonging to each variant, WT = wild type isolates.

By March 2013, the epidemic lineage became the dominant DENV strain in Singapore. It was accountable for 62.4% of all DENV strains genotyped from March 2013 to May 2014. In the present study, we analysed 744 complete *E* gene sequences and 83 whole polyprotein sequences of virus strains of the epidemic lineage detected during the study period. All isolates were classified into 12 groups (wild type and 11 additional variants) based on the fixed nucleotide substitutions in the *E* gene. The earliest isolate detected in November 2012 was considered as the wild type strain. All subsequent isolates which shared identical *E* gene sequences were considered as wild type strains (n = 239) in our mutant analysis. At *E* gene level, wild type isolates did not possess any fixed nucleotide substitutions as compared to the remaining isolates of our study population. Therefore, wild type isolates were considered as the consensus population. On the other hand, each variant possessed either single or multiple *E* gene substitutions which were uniquely fixed among members of respective variants over a period of time (Fig. 2). Each variant was named with number “13” followed by a decimal number starting from “03”. The number “13” indicated that variants belonged to the 2013 epidemic lineage. The decimal numbers from 03 to 13 were given to each variant based on the sequence of their detection.

Whole genome sequencing was carried out on randomly selected isolates (n = 83) from each group (wild type and 11 variants) in order to determine the genome wide differences during early and late phases of their detection. The number of isolates selected for whole genome sequencing from each group (Table 1) was based on the total number of *E* gene sequences detected for respective groups.

### Epidemic lineage was a mixed viral population during the initial phase of its establishment

In order to determine the genetic diversity of the epidemic lineage during its early establishment period, we analysed mutation patterns of DENV-1 genotype III isolates during the first three months (from November 2012 to January 2013) of their emergence. The analysis revealed three distinct sub-populations. The earliest sub-population which was first detected in November 2012 did not possess any substitution signature as compared to the rest of study isolates. The second sub-population possessed three fixed amino acid substitutions (prM-K90R + M-K31R + NS2A-V39I) as compared to the first sub-population. Its first occurrence among analysed samples was in December 2012. Nevertheless, both sub-populations shared identical *E* gene sequences, and therefore, were considered collectively as wild type isolates (n = 239) in our mutant spectrum analysis.

The earliest evidence of the third sub-population (named as variant 13.03) was also in December 2012. At *E* gene level, variant 13.03 (n = 29) differed from first and second sub-populations by possessing a fixed amino acid substation (E-I173V) and a synonymous substitution (T1074C). Moreover, whole genomes (n = 4) of variant 13.03 possessed a signature of three additional amino acid substitutions (NS1-N94Y + NS4B-K212R + NS5-L45M) and 18 synonymous substitutions distributed throughout the genome (Table 1). In the phylogenetic analysis, variant 13.03 formed a distinct cluster with strong posterior probability support (Fig. 1). The tMRCA analysis showed a longer period of emergence (median 3.9 yrs; 95% HPD 3.0–5.0 yrs) for the variant 13.03 than the first and second sub-populations (median 2.4 yrs; 95% HPD 2.1–2.8 yrs) (Fig. 1), indicating the likelihood of different origins for sub-populations. The presence of three genetically distinct sub-populations during the first two months of detection, therefore, indicated that the epidemic lineage was genetically mixed during the early phase of its establishment prior to the epidemic expansion.

In order to determine whether the wild type population remained as a mixed composition of first and second sub-populations throughout the epidemic, we sequenced the pre-membrane (prM) gene of 41 wild type isolates reported in each week of their existence (from November 2012 to February 2014). In addition, whole genome sequences of 16 wild type isolates (first sub-population; n = 9 and second sub-population; n = 7) were also analysed. The analysis revealed that 35 out of 57 isolates (61.4%) were of the first sub-population. The remaining 22 isolates belonged to the second sub-population. The second sub-population has been circulating as late as November 2013, suggesting the maintenance of both sub-populations until late stages of the epidemic.

### Intra-epidemic expansion of the epidemic lineage generated a mutant spectrum

In order to understand intra-epidemic evolution of the epidemic lineage, we compared complete *E* gene sequences (n = 744) generated from patient sera on a weekly basis from November 2012 to May 2014. This micro-scale analysis facilitated us to categorize isolates into mutant populations (named as variants) based on “fixed” nucleotide substitution patterns (Fig. 2) and to determine the spatiotemporal dynamics of virus diversity. Accordingly, 10 additional variants (13.04–13.13) were defined within the epidemic lineage. In general, each variant maintained the *E* gene identity even at their whole polyprotein level (Fig. 1 and Table 1). Variant 13.09 was the most common variant (n = 269) in our cohort, followed by wild type isolates (n = 239) (Fig. 2). Each of the least common variants (variants 13.07 and 13.08) included only five sequences. At *E* gene level, there were 28 “fixed” nucleotide substitutions distributed among 10 variants (13.04–13.13). Of those substitutions, only eight were non-synonymous (Fig. 2). Interestingly, five of them were founder (primary) mutations, the majority (80%, n = 4) of which emerged during early stages of the epidemic (Fig. 2). Of 20 synonymous substitutions, only seven were founder mutations and the rest were secondary (non-founder) substitutions. Four variants possessed only synonymous substitutions (Fig. 2). It is likely that the emergence of a relatively high number of variants during the early phase of the epidemic was facilitated by intense transmission of the epidemic lineage during its early establishment period.

Whole polyprotein sequence analysis of 10 variants (13.04–13.13) revealed six non-synonymous and 26 synonymous substitutions located outside the *E* gene (Table 1). This observation agreed with the previous evidence that synonymous mutations dominate the inter-host mutant spectrum of DENV populations^12^,^17^. However, our data was likely to have under-represented the true mutant spectrum of the epidemic lineage because the Sanger sequencing strategy used in our study has a high potential to capture only dominant mutants in each individual. Moreover, our genotype surveillance covered only 5–10% of the total cases reported weekly. Therefore, the actual mutant spectrum of the epidemic lineage was presumably more complex than what has been described in this article.

**Figure 3:**
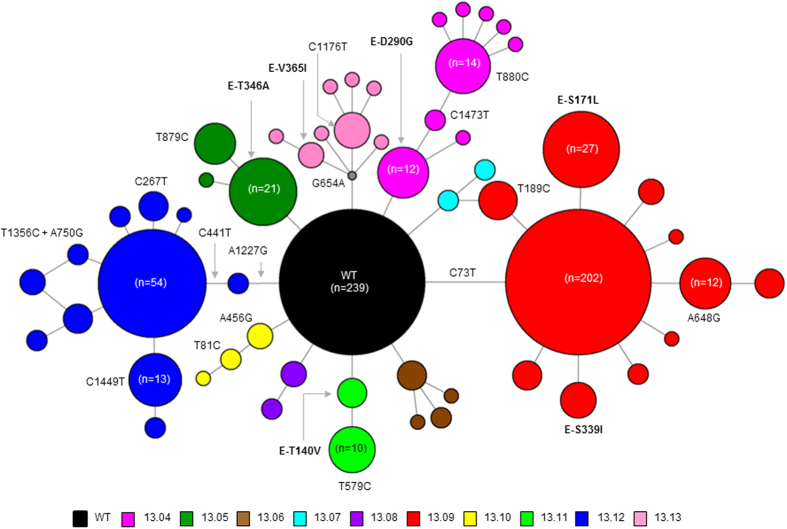
Envelope (*E*) gene based median-joining network of epidemic variants. The network was drawn in Network version 4.6.1.2 using 715 complete *E* gene sequences of DENV-1 genotype III strains generated during the epidemic. Only wild type isolates and variants 13.04–13.13, but not variant 13.03, were included in the analysis as all variants except 13.03 were shown to be originated from wild type isolates *in-situ*. All variants were colour-coded as shown in the legend. Circles represent either individual isolates or clusters. The diameter of each circle is proportional to the number of isolates within each circle. The length of lines linking circles is not proportional to the mutational distance between them. Grey node represents a hypothetical ancestral strain or a strain present in the population but not sampled.

In *E* gene-based network analysis, wild type (consensus) isolates formed the “nucleus” of the overall network of variants, illustrating the genetic connectivity of each variant (variants 13.04–13.13) to wild type (consensus) sequences (Fig. 3). Other than that, the genetic connectivity between individual variants was extremely limited. Nonetheless, genetic differences between individual variants and the wild type (consensus) sequences were subtle, indicating an extremely close genetic relatedness among variants within the epidemic lineage (Fig. 2 and Table 1). Mean pairwise distance of the closest genetic link between wild type (consensus) *E* gene sequences and those of each variant (variants 13.04–13.13) ranged from 0.1% to 0.2%. These observations testified that the epidemic lineage consisted of a spectrum of mutant viruses, characterised by a mixture of closely-related variants that displayed an extremely high genetic similarity to a consensus population. The tMRCA analysis also suggested that the earliest variant (13.04; median 2.1 yrs; 95% HPD 2.0–2.3 yrs) emerged earlier than the latest variant (13.13; median 1.5 yrs; 95% HPD 1.3–1.8 yrs), indicating the sequential emergence of 10 variants from the wild-type consensus population (median 2.4 yrs; 95% HPD 2.1–2.8 yrs). Based on the emergence, maintenance and extinction patterns of genetically distinct mutants over the epidemic period (Fig. 2), it is plausible to assume that the epidemic lineage diversity represents that of a quasispecies.

Originally theorized by Manfred Eigen and Peter Schuster in 1977^18^, a viral quasispecies is an ensemble of related but genetically distinct mutants, dominated by a set of consensus (“master”) variants that remain invariant and maintain a stable frequency in the population. Although the quasispecies concept is debatable on theoretical grounds^19^, inter- and intra-host viral diversity experiments have shown that arthropod-borne flavivirus populations such as West Nile virus (WNV)^20^ and DENV^21^,^22^ naturally exist as quasispecies. Even though restricted to inter-host data, our findings of the epidemic lineage to that consisted of a mutant swarm at a given time, therefore, support previous observations that even monophyletic flavivirus populations exist as mutant spectra in nature.

### Mutant spectrum of the epidemic lineage emerged from wild type isolates through a continuous evolutionary process

As illustrated in Fig. 2, wild type isolates were highly dominant throughout the epidemic period. The phylogenetic and network analyses revealed that 10 subsequent variants (13.04–13.13) shared the closest ancestry with wild type isolates (Figs 1 and 3). Interestingly, the mutation profiles revealed that only variants 13.06 and 13.07 were related to the second sub-population of wild type isolates whereas the remaining eight variants emerged from the first sub-population (Fig. 1). Surprisingly, none of the 10 variants (13.04–13.13) shared close identity with the third sub-population (variant 13.03), suggesting its independent existence with no evidence of further evolution.

**Figure 4:**
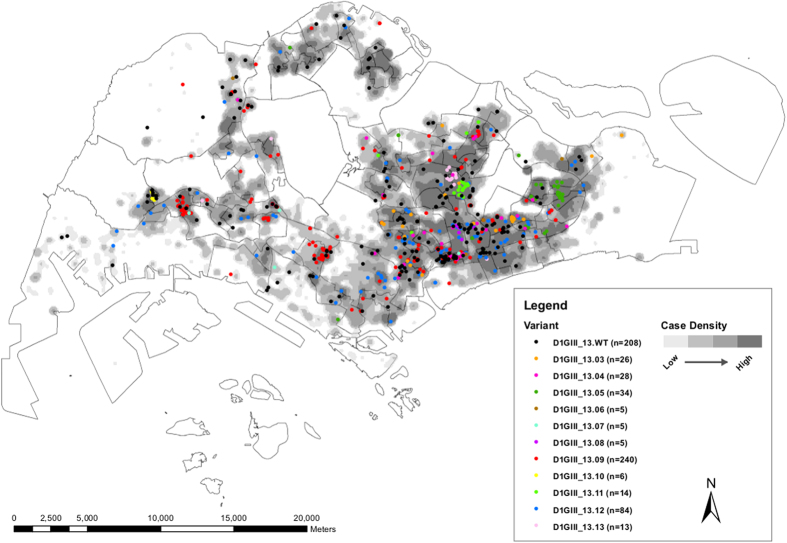
Geospatial distribution of variants of the epidemic lineage. The coordinates of reported locations available on 668 genotyped strains of epidemic lineage detected from November 2012 to end of May 2014 were mapped using the ArcGIS 10.1 ArcMap software (http://www.esri.com/software/arcgis/arcgis10/; ESRI, CA, USA). Each variant is colour-coded as shown in the figure legend. Shaded areas represent the distribution of dengue cases in a density gradient as indicated in the legend. Case density was calculated using the Kernel Density Tool in ArcGIS 10.1 ArcMap software (ESRI, CA, USA). Density values were classified into four classes, namely below 25^th^ (low in lightest tone), 26^th^–50^th^, 51^st^–75^th^ and more than 75^th^ (high in darkest tone) quantiles, using the quantile classification method. n = number of cases per variant used in the analysis.

The emergence of 10 variants (13.04–13.13) was progressive and their numbers fluctuated at a given time based on the existence and elimination patterns (Fig. 2). While several variants were long-circulating, some became extinct quickly. Those long-circulating variants (13.04, 13.09 and 13.12) were generally dominant and widely distributed (Figs 2 and 4). They accumulated non-founder (secondary) substitutions in a classic step-ladder pattern characteristic of *in-situ* evolution (Fig. 2). *E* gene based analysis revealed that the mean mutation rate of the epidemic lineage (1.33 × 10^−3^ substitutions per site per year; 95% HPD 9.75 × 10^−4^–1.72 × 10^−3^) to be approximately as twice as the average rate reported previously for DENV-1 *E* gene sequences^23^, suggesting that DENVs potentially evolve faster than empirical rates during epidemics.

The appearance of secondary substitutions was rapid during periods of intense transmission that presumably allow viruses to replicate fast. For example, variant 13.09 acquired three non-synonymous (E-S171L, E-T339I and NS4B-S243T) and four synonymous (E-A1488G, prM-C354T, NS2A-A3783G and NS4B-A6537G) substitutions within a 3-week period in April 2014 during which variant 13.09 population expanded and subsequently accumulated 75.1% (n = 202) of its analysed sequences. Similar observations were also made with regard to other dominant variants. Interestingly, despite having relatively a high number of mutations, dominant variants acquired only a few amino acid substitutions as compared to wild type isolates (Fig. 2). They were mainly non-founder substitutions (Fig. 2).

In contrast to dominant variants, “low-profile” variants (13.05, 13.06, 13.07, 13.08, 13.10, 13.11 and 13.13) were less common (Fig. 2) and were generally short-lived. Unlike dominant variants, less-dominant variants possessed amino acid alterations as founder substitutions (Fig. 2 and Table 1). Interestingly, “low-profile” variants such as 13.05, 13.10 and 13.11 generally showed localized transmission and tend to disappear with the successful control of respective case clusters. Given the well-developed transportation network and a highly mobile population in Singapore, it was highly likely that those less-dominant variants moved out of the cluster areas, but were unable to establish wide scale transmission.

**Figure 5:**
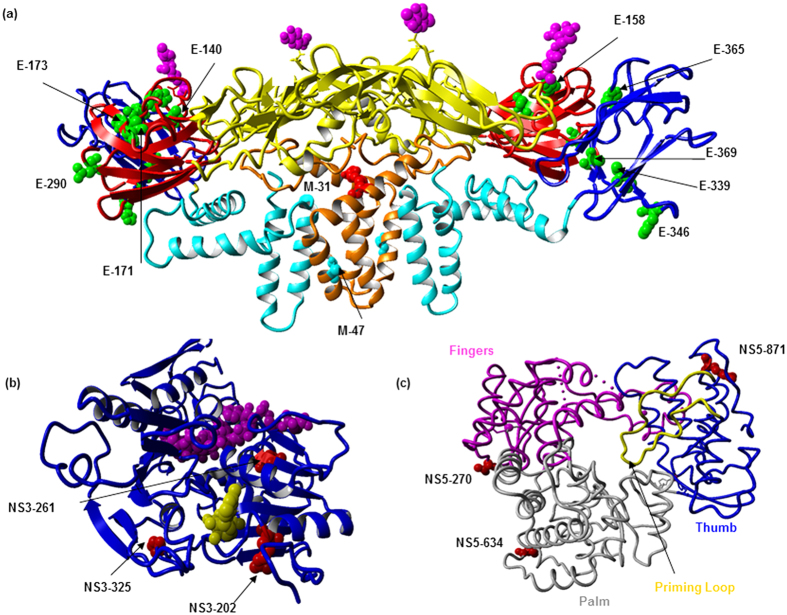
Crystal structures of E, M, NS3 and NS5 proteins showing substituted positions. (**a**) X-ray crystal structure (PDB: 3J2P) showing three domains of the E and M proteins. ED1 in red, EDII in yellow and EDIII in blue ribbons, E transmembrane in cyan. Substituted positions 140, 158, 171, 173, 290, 339, 346, 365, 369 in the E protein are colored in green. Positions 31 and 47 from the M protein (in orange ribbon) are colored in red and cyan, respectively. (**b**) NS3 protein (PDB: 2JLV) in complex with ssRNA (highlighted in magenta) and 5′-adenylyl-β, γ-imidodiphosphate (AMP-PNP). Positions 202, 261 and 325 are highlighted in red. (**c**) The NS5 protein (PDB: 4C11) RNA-dependent RNA polymerase (RdRP) domain. The RdRP palm is in gray, fingers in magenta, thumb in blue, and the priming loop is in yellow. Substituted positions 270, 634, 871 are highlighted in red.

These findings suggested that variants structurally identical to the wild type isolates were likely to have a survival advantage over those with structural differences. The fleeting existence of “low-profile” variants may, therefore, be due to the fitness cost incurred by structural deviations from the consensus sequence. As shown in Fig. 2 and Table 1, amino acid substitutions among variants were predominantly observed in *E* gene sequences. Having exposed on the surface of virus particles, *E* gene domains (EDI, EDII and EDIII) are primarily targeted by immune mediators^24^,^25^,^26^ and are subjected to adaptive changes to evade host immune pressure. Except for E-T346A substitution (low-profile variants 13.06 and 13.07) which is adjacent to an antibody binding domain (PDB: 4FFY), none of the *E* gene non-synonymous substitutions observed in our study isolates were in known structural determinants of antibody neutralization. Nevertheless, many of them were found to reside within known T-cell and B-cell epitope regions (Table 2). Low-profile variants 13.06 and 13.07 also possessed additional non-conservative (E-T369I and NS5-I272T) and conservative substitutions (prM-K90R, M-K31R and NS2A-V39I) which had potential structural and immune interaction implications (Fig. 5 and Table 2).

Assuming that those structural and immune implications could have been deleterious to respective variants, in the presence of a relatively high mutation rate, relatively small populations of “low-profile” variants may have been subjected to a “Muller’s rachet” effect^27^, in which deleterious mutations potentially exposed them to bottleneck events. Severe bottleneck events have previously been shown to cause fitness losses in RNA virus populations^28^. On the other hand, the localized presence of “low-profile” mutants in certain localities could be due to the effect of competitive exclusion phenomenon^29^, in which stochastic emergence of highly advantageous mutants could lead to an exclusion of other mutants^30^. Alternatively, amino acid substitutions in “low-profile” mutants could have disrupted the fitness trade-off that allows arboviruses to survive in alternating vertebrate and invertebrate hosts. Previous evidence on WNV^31^ and DENV^32^ populations indicates that mutant spectra and trade-off patterns are host-dependent, although the absolute necessity of a trade-off for viral fitness in alternative hosts is debatable^32^,^33^. It is noteworthy that structural differences were not only restricted to “low-profile” variants. Dominant variants such as 13.04 (E-D290G), 13.09 (E-S171L, E-T339I and NS4B-S243T) and 13.12 (NS3-A325S) also possessed the amino acid substitutions that were potentially implicated in the structural stability and immune interactions (Table 2). Although synonymous substitutions do not result in changes in the biochemical properties of the translated protein, they have been shown to exhibit codon usage bias in DENV populations^5^, to contribute to host adaptation and pathogenicity in other viruses^34^,^35^ and to affect the fitness of RNA viruses, especially ssRNA viruses, *in vitro*^36^. Therefore, it is worthwhile to investigate whether the observed genetic differences and their epistatic interactions imposed a fitness cost, especially on less-dominant variants, in both human and vector hosts and consequently wiped them out from circulation as part of the purifying selection.

**Figure 6:**
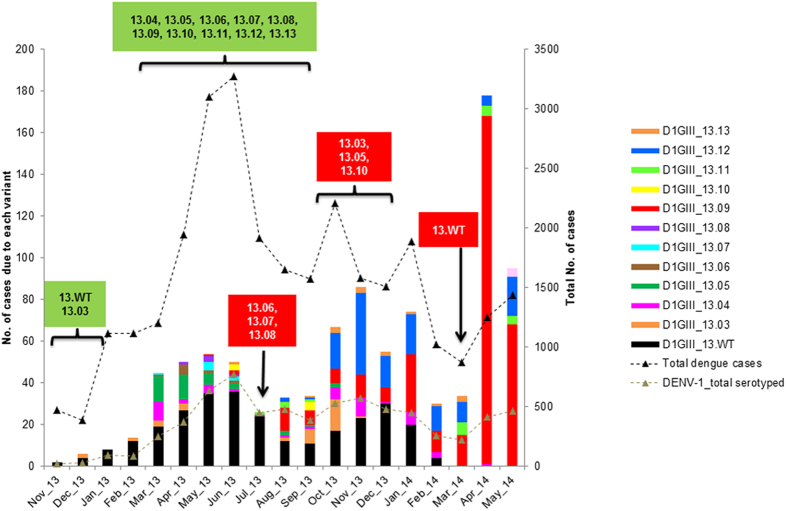
Correlation between viral diversity and disease transmission. The figure shows the comparison of temporal fluctuation between the number of variants and total number of cases on a monthly basis. The analysis included respective data collected from November 2012 to May 2014.

The majority of non-synonymous differences observed in the epidemic lineage were evolutionarily neutral, even though several residues were under episodic diversifying selection (Table 3). This general neutrality of the evolutionary process suggested a potential role for genetic drift in extinction events, especially of “low-profile” variants. Low-profile variants such as 13.06, 13.07 and 13.08 became extinct during a genetic bottleneck that coincided with low case incidence in mid-2013 (Fig. 6). It is known that the random effects of genetic drift are more pronounced during bottleneck events in small populations than in large populations^37^. Furthermore, the less-dominant nature of variants 13.08 and 13.10 that shared a structural resemblance to wild type isolates suggested that the existence and extinction patterns of variants may have also been driven by stochastic forces related to non-inherent factors such as human movement, human density, population immunity, differences in vector density and competence, availability of breeding opportunity and the limited flight range of *Aedes* mosquitoes in respective locations. This observation is consistent with previous evidence on the stochastic nature of extinction and replacement of DENV-1 lineages during inter-epidemic periods^38^.

### Temporal dynamics of the mutant spectrum correlated with transmission intensity

**Figure 7:**
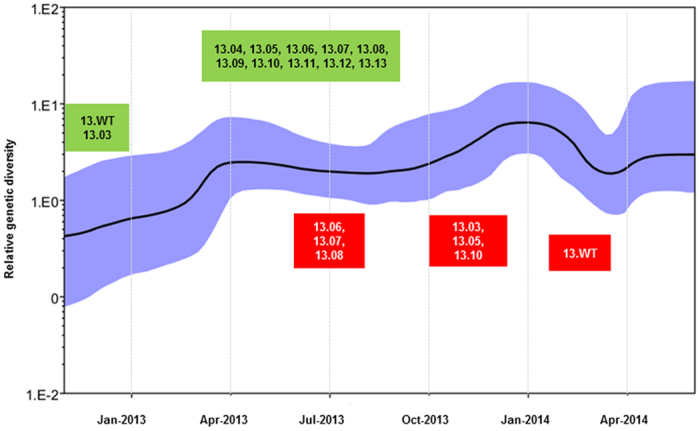
Temporal variation of virus diversity and emergence/disappearance of variants. Bayesian skyline plot was generated in the BEAST package v1.7.4^51^ using a TN93+I+G substitution model together with an uncorrelated log-normal relaxed molecular clock and a Bayesian skyline coalescent model. One hundred million generations of MCMC chains were run with sampling at every 10,000 generations. The black line illustrates the fluctuations in median genetic diversity over time and the shaded area represents 95% highest posterior density (HPD) values. Green and red boxes indicate the time of emergence and extinction of variants included within each box respectively. D1GIII = DENV-1 genotype III; n = number of sequences per each variant used in the analysis; WT = wild type.

In order to determine the relationship between mutant diversity and disease transmission, we compared fluctuations of the number of variants and total number of dengue cases on a monthly basis in 2013 (Fig. 6). In this context, we considered number of cases (total reported and total DENV-1) as a proxy for the intensity of virus transmission. In addition, we constructed a Bayesian skyline plot to visualize longitudinal variations of the genetic diversity of epidemic lineage in 2013 (Fig. 7). The analysis showed that number of DENV-1 cases and overall case incidence due to all four serotypes were positively correlated (r = 0.87, p < 0.001). Based on island wide case surveillance data that includes cases reported by EHI diagnostics as well as other laboratories, DENV-1 consisted of 65.4% (n = 6971) of the total number of serotyped cases (n = 10653, 36.4% of total reported cases) from November 2012 to May 2014, indicating its dominant role during the epidemic. Our genotype data also showed that 89.1% of DENV-1 cases detected during the same period were due to the epidemic lineage (Supplementary Table S1), further narrowing down the epidemic attribution to our study lineage. There was a stronger correlation between the temporal diversity of epidemic lineage and DENV-1 cases (r = 0.78, p < 0.001) than that of the total number of cases (r = 0.71, p < 0.001). Nonetheless, both relationships were statistically significant. Bayesian skyline plot also showed an agreement between the longitudinal variation of genetic diversity of epidemic lineage and dengue cases (Fig. 7).

We observed that inclination of genetic diversity preceded the increase in actual reported cases. Similar observations have previously been made in a long-term longitudinal study conducted by Bennett and co-authors on DENV-4 populations in Puerto Rico^13^. This was obvious in March 2013, at the beginning of the first epidemic wave that occurred from March to June 2013 (Fig. 6) during which all 10 variants (13.04–13.13) described in the present study emerged. However, the epidemic lineage went through two bottleneck events in July 2013 and in February-March 2014 (Figs 6 and 7). Coincidentally, dengue incidence also dropped to its lowest level during the epidemic. The low case incidence subsequent to two epidemic peaks may be a consequence of intense vector control efforts in response to the case burden. The temporary suppression of the vector population may have brought down the transmission intensity, leading to bottleneck events that lead to the extinction of multiple variants (Fig. 6). The impact of immune pressure in the human in restricting the variant repertoire is also important as the immune-driven selection is known to occur even during short-term, acute dengue infection^39^ and is hypothesized to be a major driver of viral diversity^40^. Despite the lack of data pertaining to bottleneck events in natural mosquito-borne viral populations during epidemics, the experimental evidence has shown that human-vector transmission cycle imposes severe bottlenecks in mosquito-borne RNA virus populations^41^,^42^, including DENV^40^. Only a small proportion of the viral mutational spectrum, mainly the consensus population and dominant variants, is likely to be successful during such bottlenecks^40^,^43^. Though detrimental to many non-dominant variants, the human-vector transmission allows new DENV variants to emerge^40^. This was obvious during the first bottleneck event in July 2013 during which mainly the wild type variants survived (Fig. 6). The subsequent recovery phase saw the emergence of two dominant variants (13.09 and 13.12) at the expense of more less-dominant variants (Fig. 6). Despite the extinction of even the wild-type variants, those two dominant variants survived the second bottleneck event in February–March 2014. Their emergence and expansion resulted in an increase in case incidence after each bottleneck event (Fig. 6 and Supplementary Video S1). These observations indicated that the pattern of emergence and extinction of mutants fluctuated with transmission intensity of viral strains. Indeed, the transmission intensity of rapidly-evolving RNA viruses shows a direct relationship with viral population sizes and, therefore, is one of the important drivers of their evolutionary dynamics^44^.

Based on these findings, we presumed a higher density of variants to be found in transmission “hot spots” than in low-transmission areas. A previous study has also shown relatively high genetic diversity of WNV in a “hot spot” of arboviral transmission in USA^45^. Accordingly, we mapped postal code information of respective cases (n = 668) using the ArcGIS 10.1 ArcMap software (ESRI, CA, USA) to determine the distribution of individual variants against that of the total number of DENV-1 cases. As illustrated in Fig. 4 and demonstrated in Supplementary Video S1, cases were mainly clustered in Central and Southeast regions of the country. During initial phase of the epidemic, intense transmission was observed along a southeast belt. Cases gradually spread northwards to the centre before establishing in the Western part of the country, especially during the latter half of 2013. Interestingly, spatial distribution of virus variants also followed the same pattern (Fig. 4). The majority of variants were distributed in Southeast and Central parts of the island where the highest number of cases was reported. As postulated, spatial distribution pattern of genotyped cases showed aggregation of relatively a high number of variants in transmission hot-spots, supporting the earlier notion that intense transmission was likely to have driven the mutant diversity of epidemic lineage.

In conclusion, our observations suggested that the mutant spectrum of epidemic lineage was subjected to a rapid and consistent evolutionary process *in-situ*. The presence of episodic diversifying forces selecting for a repertoire of novel substitutions (Table 3) indicated that this evolutionary process was distinct but transient^46^. Differential dynamics of variants with presumably the same fitness advantage indicated a stochastic nature of this evolutionary process. Moreover, the resulting mutant diversity intensified during periods of high transmission, implying a relationship between the force of *in-situ* evolution and transmission intensity during the epidemic. These observations indicated that tracking genetic diversity through time is a useful tool to infer DENV transmission dynamics and to complement surveillance data in a risk assessment perspective to support disease control operations. Our findings also suggested that cross-sectional surveys and longitudinal virus surveillance efforts with limited sample sizes tend to underestimate the true diversity of viral populations. Such information is salient to understand the viral basis of variations in clinical outcome and immune response which is a prerequisite of effective vaccine design.

## Methods

### Collection of Dengue virus positive sera

The diagnostics section of Environmental Health Institute (EHI) routinely receives blood samples obtained from dengue suspected patients through an island wide network of hospitals and general practitioners. For the present study, samples received with patient consent from November 2012 to May 2014 were included (Supplementary Table S1). DENV infection was confirmed by using the SD Bioline Dengue Duo kit (Standard Diagnostics INC., South Korea). All NS1 positive samples were subjected to serotyping as described below. All consented DENV positive samples were used for genome sequencing as part of the existing virus surveillance programme at EHI.

### Ethics statement

All DENV-positive sera used for the genome sequencing were collected after obtaining the written informed consent from respective patients. All experimental protocols were conducted in accordance with the approved guidelines approved by the Institutional Review Board of National Environmental Agency, Singapore (IRB003.1).

### Virus isolation

Virus strains were isolated from acute-phase sera as described elsewhere^47^. A maximum of three passages was done for each sample based on the original virus load.

### Determining the serotype of Dengue virus

Viral RNA was extracted from sera using the QIAGEN QIAamp viral RNA mini kit (QIAGEN, Hilden, Germany) according to manufacturer’s recommendations. The complementary DNA (cDNA) was synthesized using extracted RNA as described before^47^. DENV serotypes were determined by using a real-time PCR assay as described in detail elsewhere^47^,^48^. A serotype-specific conventional semi-nested PCR assay was performed on sera with low virus titres using a protocol described previously^47^. Amplified products were visualized in 2% agarose gels stained with GelRed (Biotium Inc., USA).

### Whole genome and envelope (*E*) gene sequencing of Dengue virus type 1

*E* gene sequences were generated directly from patient sera, whereas whole genome sequencing was performed on strains isolated from respective patient sera. Samples for whole genome sequencing were selected to represent different groups of viruses (variants) described in the article. Genomes of at least two isolates detected during early and late phases of their existence were fully sequenced.

The protocol for the complete *E* gene (~1.5 kb) sequencing has been described before^47^. Amplification and sequencing primers were designed to cover the entire genome of DENV-1 in overlapping fragments (Supplementary Table S2). Additional two sets of primers were used to capture 5′ and 3′ untranslated regions (UTRs) of DENV-1 genome^47^. PCR amplification of each fragment was performed as described in Supplementary Table S2. Amplified products were purified using Expin PCR SV mini kit (GeneAll Biotechnology, Korea) according to manufacturer’s instructions. Sequencing of purified PCR products was performed by a commercial sequencing company (AITbiotech Pte Ltd, Singapore) according to the BigDye Terminator Cycle Sequencing kit (Applied Biosystems, USA) protocol.

### Assembly and analysis of sequences for genetic differences

Raw nucleotide sequences were assembled using the Lasergene package version 8.0 (DNASTAR Inc., Madison, WI, USA). Contiguous sequences were aligned in BioEdit v7.0.5 software suite^49^. Comparison of nucleotide and amino acid sequences deduced from nucleotide alignments was performed by using the BioEdit v7.0.5 software suite^49^. The comparative analysis included 1783 complete *E* gene and 1504 polyprotein sequences of DENV-1 isolates available in Genbank database. The pairwise distance (p-distance) between wild-type strains and individual variants described in this study was calculated using MEGA 6 software package^50^. A summary of the number of sequences used in different types of analysis is given in Supplementary Table S3.

### Phylogenetic analyses of genome sequences of Dengue virus type 1 genotype III

The whole-polyprotein based maximum clade credibility tree was constructed using the Bayesian Markov Chain Monte Carlo (MCMC) method implemented in the BEAST package v1.7.4^51^. Based on jModeltest 2.1.5^52^, the general time reversible model with gamma distribution and invariant sites (GTR+G+I) was used together with an uncorrelated log-normal relaxed molecular clock model. The year of origin of each isolate was included for the calculation of the time to most recent common ancestor (tMRCA) of different lineages, including the epidemic lineage. The dataset included 38 whole polyprotein sequences generated in this study and 44 DENV-1 sequences retrieved from GenBank database. Of 83 whole genomes generated, only 38 sequences representative of each variant were included in the phylogenetic analyses to minimize overcrowding.

Bayesian skyline plot for genetic diversity of epidemic strains was constructed based on *E* gene sequences (n = 291), using the Bayesian Markov Chain Monte Carlo (MCMC) method implemented in the BEAST package v1.7.4^51^. Substitution rate of epidemic strains was also calculated during the same analysis. *E* gene sequences were selected to represent each variant on a weekly basis from November 2012 to May 2014. As predicted by jModeltest 2.1.5^52^, the Tamura and Nei 1993 substitution model (TN93+G+I) was used together with an uncorrelated log-normal relaxed molecular clock model and a Bayesian skyline coalescent model.

In all Bayesian MCMC analyses, 100 million generations of MCMC chains were run with sampling at every 10,000 generations. The MCMC output (including Bayesian skyline plot for genetic diversity) was analysed using the Tracer Version 1.5 programme (http://tree.bio.ed.ac.uk/software/tracer/). The uncertainty in parameter estimates was expressed as 95% highest posterior density (HPD). All runs of MCMC were repeated to ensure convergence with a minimum effective sample size (ESS) of >200.

### Median joining network analysis

The median joining network (Fig. 3) was used to visualize fine-scale genetic relationships based on single nucleotide polymorphisms (SNPs) in analysed sequences. The network was constructed using the Network version 4.6.1.2 software^53^. The presented network was constructed using complete *E* gene sequences (n = 715) of DENV-1 genotype III isolates. Variant 13.03 (n = 29) was not included in the Network analysis as the whole genome analysis revealed that subsequent variants (13.04–13.13) were unlikely to have descended from 13.03.

### Prediction of structural implications and immune interactions

Protein Data Bank (PDB) structures 3J2P^54^ which illustrates the M and E protein heterotetramer, 2JLV^55^ which represents the NS3 protein (serine protease as well as RNA helicase and RTPase/NTPase) and 4C11 showing the NS5 protein were downloaded from the Research Collaboratory for Structural Bioinformatics (RCSB) protein data bank and visualized using YASARA^56^. Changes to protein stability were estimated using Fold-X^57^. PDB structures 3G7T, 4FFY, 3J2P, 4O6B, 2JLV, 4CTJ, 4C11 were used and minimized with the RepairPDB function. Substitutions were introduced separately or in combinations assuming they were to co-occur naturally. This minimization procedure was repeated five times and the averages were taken as the final predicted free-energy changes.

Dengue immune epitopes derived from B-Cell and T-Cell assays were downloaded from Immune Epitope Database and Analysis Resource (IEDB) (www.iedb.org, sourceOrgId 11053), and the epitopes were mapped to the DENV-1 polyprotein using blastp (www.blast.ncbi.nlm.nih.gov/Blast.cgi).

### Selection pressure analyses

Whole polyprotein sequences (n = 83) were subjected to the selection pressure analysis to determine residues under selection. Genome-wide dN/dS ratios were computed in Datamonkey web server^58^ using the single likelihood ancestor counting (SLAC), fixed effects likelihood (FEL), internal fixed effects likelihood (IFEL) and the mixed effect model of evolution (MEME) methods. The Muse and Gaut (MG94) codon-based substitution model, GTR nucleotide substitution bias model and neighbour joining phylogeny were used as analytical parameters available in the Datamonkey web interphase. Significance levels were set at p = <0.05.

### Spatial analysis using Geographical Information System

Personal identifiers of consented cases were removed in order to protect the privacy of patients. The postal codes of locations that were epidemiologically confirmed as places of acquiring the infection by each individual were used to plot the spatial distribution of DENV 1 genotype III sequences (Fig. 4). The analysis compared the location information of 668 *E* gene sequences (classified into variants) used in this study and the total number of cases with location information reported by the national dengue surveillance programme from November 2012 to May 2014. Two datasets were prepared in two layers and were subsequently overlaid to visualize the spatial positioning of individual variants in the background of overall case distribution. For the total number of reported cases, a density gradient was calculated using the Kernel Density Tool in ArcGIS 10.1 ArcMap software (http://www.esri.com/software/arcgis/arcgis10/; ESRI, CA, USA). Density values were classified into four classes, namely equal or less than 25^th^ (low), 26^th^–50^th^, 51^st^–75^th^ and more than 75^th^ (high) quantiles, using the quantile classification method, so that higher densities were shown in darker tones of gray and lower densities in lighter tones. The same analysis was repeated in R software package version 2.15.0 (http://www.R-project.org/)^59^ for the supplementary video (Supplementary Video S1), but on a weekly basis, in order to demonstrate the cumulative pattern of case and variant distribution on a longitudinal scale.

### Statistical analysis

To determine the correlation between the diversity of epidemic lineage and transmission intensity, we compared the number of DENV-1 genotype III variants against the total number of DENV-1 cases and total number of reported cases on a monthly basis from November 2012 to May 2014. We considered number of reported cases as a surrogate indicator of transmission intensity. The strength of correlation was quantified using Pearson’s correlation coefficient in R software package version 3.0.1 (http://www.R-project.org/). An alpha value of 0.05 was used to determine the statistical significance of p-values.

## Additional Information

**Accession codes**: Sequences used in the phylogenetic analysis were deposited in GenBank database under the accession numbers KM403575-KM403636.

**How to cite this article**: Hapuarachchi, H. C. *et al.* Intra-epidemic evolutionary dynamics of a Dengue virus type 1 population reveal mutant spectra that correlate with disease transmission. *Sci. Rep.* **6**, 22592; doi: 10.1038/srep22592 (2016).

## Supplementary Material

Supplementary Information

Supplementary Video S1

## Figures and Tables

**Table 1:** Whole genome (except *E* gene) analysis of fixed substitutions distributed among variants

Polyprotein position	Gene	Gene position	Wild type (Ref*)	Wild type (epidemic lineage)	Variants (Epidemic lineage)	Variant name
**Non-synonymous substitutions**						
204	prM	90	K	K/R	R	13.06 (2/2), 13.07 (2/2)
236	prM	122	K	K/R	R	13.06 (2/2), 13.07 (2/2)
869	NS1	94	S	N	Y	13.03 (4/4)
1166	NS2A	39	V	V/I	I	13.06 (2/2), 13.07 (2/2)
1800	NS3	325	A	.	S	13.12 (16/18)
2456	NS4B	212	K	.	R	13.03 (4/4)
2487	NS4B	243	S	.	T	13.09 (9/17)
2538	NS5	45	L	.	M	13.03 (4/4)
2763	NS5	270	V	I	T	13.06 (2/2)
**Synonymous substitutions**						
354	prM	12	C	.	T	13.09 (9/17)
480	prM	138	G	.	A	13.04 (4/6)
483	prM	141	C	T	.	13.11 (3/4)
760	prM	418	C	T	.	13.03 (4/4)
2865	NS1	540	C	.	T	13.06 (2/2)
3339	NS1	1014	C	T	.	13.13 (6/6)
3783	NS2A	402	G	A	.	13.09 (9/17)
3798	NS2A	417	T	.	C	13.12 (16/18)
3864	NS2A	483	G	.	A	13.13 (5/6)
4083	NS2B	48	T	.	C	13.04 (4/6)
4167	NS2B	132	C	T	.	13.03 (4/4)
4284	NS2B	249	C	T	.	13.13 (6/6)
4560	NS3	135	G	.	A	13.04 (5/6)
4770	NS3	345	C	.	T	13.12 (16/18)
4857	NS3	432	A	G	.	13.03 (4/4)
5367	NS3	942	C	T	.	13.13 (6/6)
5487	NS3	1062	T	C	.	13.03 (4/4), 13.06 (2/2), 13.07 (2/2)
5637	NS3	1212	C	.	T	13.08 (2/2)
5688	NS3	1263	C	T	.	13.03 (4/4)
5757	NS3	1332	A	G	A	13.03 (4/4)
5760	NS3	1335	C	.	T	13.03 (4/4)
6261	NS3	1836	A	.	G	13.13 (5/6)
6537	NS4A	255	A	.	G	13.09 (9/17)
6600	NS4A	321	G	.	A	13.03 (4/4)
6639	NS4A	360	T	.	C	13.03 (4/4)
7098	NS4B	369	T	C	.	13.13 (6/6)
7176	NS4B	447	C	A	G	13.09 (13/17)
7367	NS4B	638	A	.	G	13.03 (4/4)
7482	NS5	9	C	T	.	13.03 (4/4)
7596	NS5	123	T	C	.	13.03 (4/4)
7779	NS5	306	G	A	.	13.03 (4/4)
7833	NS5	360	C	T	.	13.04 (5/6)
8034	NS5	561	T	.	C	13.03 (4/4)
8139	NS5	666	T	.	C	13.05 (4/4), 13.08 (2/2), 13.09 (17/17), 13.10 (2/2), 13.11 (4/4), 13.12 (18/18), 13.13 (6/6)
8288	NS5	815	T	.	C	13.06 (2/2)
8655	NS5	1182	C	.	T	13.03 (4/4)
8946	NS5		C	.	T	13.03 (3/4)
9186	NS5	1713	G	.	A	13.03 (4/4)
9627	NS5	2154	T	.	A	13.09 (15/17)
9789	NS5	2316	C	.	T	13.04 (4/6)
10050	NS5	2577	C	.	T	13.10 (2/2)
10104	NS5	2631	T	C	.	13.03 (4/4)
10143	NS5	2670	C	.	T	13.11 (4/4)

Substitutions which are fixed in more than 50% of isolates sequenced for each variant are shown. Fraction in parenthesis represents number of strains with the indicated substitution against the total number of whole genomes of the variant. The table does not list *E* gene substitutions which are shown in Fig. 2. *Reference sequence = NCBI Accession No. NC001477.

**Table 2:** Structural significance and immune interactions of non-synonymous substitutions detected in the epidemic lineage

Protein	Substitution at protein level	Substitution at polyprotein level	Predicted structural significance and immune interactions
Capsid (C)	L105F	L105F	No relevant structure found at this position; Resides within a known T cell epitope (IEDB identifiers 184458 and 184847)
Pre-membrane (prM)	K90R	K204R	Resides in the furin cleavage site “R-X-R/K/X-R”^60^,^61^; The majority of strains has “RRDKR” at positions 87–91. Hence, the mutation allows furin to recognize not just the “RDKR” (88–91) as cleavage site, but could also recognize “RRDR” (87–90) as an alternative cleavage site.
Membrane (M)	L47F	L252F	Resides in a trans-membrane region; Effects of substitution are uncertain; Resides within a known T cell epitope (IEDB identifier 183595)
	K31R	K236R	Position 31 lies in an amphipathic α-helices (stem anchor) region which is half buried in the outer lipid leaflet. Residue in close vicinity to R/K31 has been shown to play a role in viral assembly and maturation^62^; Substitution could enhance packing and have some effects during conformational change of E protein.
Envelope (E)	S171L	S451L	A semi-buried residue in EDI; Resides within a known T cell epitope (IEDB identifier 186048); FoldX stability calculations suggested this substitution to be stabilizing
	H158Q	H438Q	Resides less than 4 Angstrom from N-glycosylated N153 in EDI; N-glycosylation at site 153 has been shown to affect DENV-2 infectivity
	T339I	T619I	A buried residue within EDIII; Resides within a known discontiguous B cell epitope (IEDB identifier 176781); Fold-X stability calculations suggested this substitution to be stabilizing
	V365I	V645I	A buried residue in EDIII; effects of substitution are likely to be small; Fold-X stability calculations suggested this substitution to be mildly stabilizing
	T369I	T659I	A buried residue in EDIII; Fold-X stability calculations suggested this substitution to be stabilizing
	T346A	T626A	Lies within 5 Angstrom from bound antibody (PDB: 4FFY); Resides within a known discontiguous B cell epitope (IEDB identifier 176781)
	I173V	I453V	A buried residue in EDI; Resides within a known T-cell (IEDB identifier 186048) and a discontiguous B cell epitope (IEDB identifier 173906); Fold-X stability calculations suggested this substitution to be destabilizing
	I140V	I420V	A buried residue in EDI, effects of substitution are likely to be small.
	D290G	D570G	Resides on EDI prior to EDI-DIII loop; Possibly play a role when the hinge (EDI-DIII loop) opens up. Fold-X stability calculations suggested that this substitution stabilizes the pre-fusion E protein structure^£^
Non-structural (NS) 1	N94Y	N869Y	Resides within an exposed region; Resides within known B cell epitopes (IEDB identifiers 132320 and 132267); Fold-X stability calculations suggested this substitution to be mildly stabilizing
	T128I	T903I	Resides within a disordered loop region with unknown function; Resides within a known B cell epitope (IEDB identifier 132152)
NS2A	V39I	V1166I	NS2A-V39I lies in a predicted transmembrane segment that may peripherally interact with the ER membrane on the lumen side, without spanning the lipid bilayer^63^. The conservative Val to Ile substitution is unlikely to have any drastic effects on the peripheral interaction of this segment with the ER lipid bilayer. Resides within a known T cell epitope (IEDB identifier 225402).
NS3	R202K	R1677K	Resides close to AMP/ADP binding site; effects of R->K are unknown
	M261I	M1736I	Located in the ssRNA binding site; Likely to have effects on the helicase activity; Resides within known T cell epitopes (IEDB identifiers 150166 and 24915); Fold-X stability calculations suggested that this substitution destabilizes the NS3 protein
	A325S	A1800S	Lies at an allosteric site i.e. more than 13 Angstrom from the NTPase/RTPase binding site; Unlikely to have a direct effect on its helicase or NTPase/RTPase function; Fold-X stability calculations suggested this substitution to be mildly stabilizing
NS4B	K212R	K2456R	No relevant structure found; Resides within known T cell epitopes (IEDB identifiers 150579 and 150666)
	S243T	S2487T	Lies at the C-terminal end of the TMD5 domain of NS4B; This region has been shown to be involved in the homodimerization of NS4B which played an important role in the viral replication^64^, and is also involved in the suppression of the host RNAi defense mechanism^65^.
NS5	I270T	I2763T	This position falls between the linker between methyltransferase domain and the RdRp domain and has been shown to play a role in viral replication. A mutation from Lys to Ala at this position is shown to decrease polymerase activity in DENV4^66^. A non-conservative substitution at this equivalent position may possibly also affect polymerase activity.
	L45M	L2538M	Located close to the putative active site; Substitution could affect packing, and hence alter efficiency of transferase activity
	E634G	E3127G	Located at an exposed region of RdRP; Fold-X stability calculations suggested this substitution to be mildly stabilizing
	R871K	R3364K	Located at an exposed region of RdRP; Effects of substitution are unknown

In FoldX stability calculations, substitutions with ddG >−2 and < = −0.5 are labelled as mildly stabilizing, while those with ddG > = 0.5 and <2 are labelled as mildly destabilizing. Substitutions with ddG < = −2 or > = 2 were labelled as stabilizing and destabilizing respectively. ^£^FoldX stability calculations derived from less reliable Cryo-EM structure.

**Table 3:** Genome-wide selection pressure analysis of non-synonymous substitutions detected in the epidemic lineage.

Substitution	Other reports of similar substitution	Selection pressure analysis method			
		SLAC	FEL	IFEL	MEME
PrM-K90R	Vietnam (GQ199833), Cambodia (FJ639678)	Neutral	Negative (p = 0.04)	Neutral	Neutral (p = 0.09)
prM-K122R	Common mutation in isolates from Southeast Asia and the Americas	Neutral	Neutral (p = 0.07)	Neutral	Neutral (p = 0.08)
E-I140V	Unique	Neutral	Neutral (p = 0.79)	Neutral	Neutral (p = 0.60)
E-S171L	Unique	Neutral	Neutral (p = 0.51)	Neutral	Neutral (p = 0.30)
E-I173V	Unique	Neutral	Neutral (p = 0.79)	Neutral	Neutral (p = 0.60)
E-D290G	Unique	Neutral	Neutral (p = 0.38)	Neutral	Positive (p = 0.02)
E-S339I	China (KF864667), Angola (KF184975), India (JQ922546), India (JN903578), Singapore (EU081258), Cote D’Ivoire (AF298807)	Neutral	Neutral (p = 0.19)	Neutral	Neutral (p = 0.39)
E-T346A	Unique	Neutral	Neutral (p = 0.75)	Neutral	Neutral (p = 0.67)
E-V365I	AF226686, Solomon Islands (JQ655094), Japan (AB204803)	Neutral	Neutral (p = 0.13)	Negative (p = 0.03)	Positive (p = 0.05)
E-A369I	Unique	Neutral	Neutral (p = 0.15)	Neutral	Neutral (p = 0.67)
NS1-S94Y	Unique	Neutral	Neutral (p = 0.69)	Neutral	Neutral (p = 0.67)
NS2A-V39I	Unique	Neutral	Neutral (p = 0.34)	Neutral	Positive (p = 0.01)
NS3-A325S	Unique	Neutral	Neutral (p = 0.23)	Neutral	Positive (p = 0.04)
NS4B-K212R	India (JQ917404, KF289072, JQ692085)	Neutral	Neutral (p = 0.81)	Neutral	Neutral (p = 0.33)
NS5-L45M	Unique	Negative (p = 0.02)	Negative (p = 0.001)	Neutral	Neutral (p = 0.67)
NS5-V270T	Unique	Neutral	Negative (p = 0.05)	Neutral	Positive (p = 0.05)

^*^Reference sequence = NCBI Accession No. NC001477. The cut-off for significance level was set at 0.05. prM = membrane protein, E = envelope protein, NS = non-structural proteins.
