# Supplementary material for: Intra-epidemic evolutionary dynamics of a Dengue virus type 1 population reveal mutant spectra that correlate with disease transmission
Source: Sci Rep. 2016 Mar 4;6:22592. doi: 10.1038/srep22592 (PMC4778070; doi:10.1038/srep22592)
Supplement: Supplementary Information [file srep22592-s1.pdf]

Intra-epidemic evolutionary dynamics of a Dengue virus type 1 population reveal mutant spectra that correlate with disease transmission

Hapuarachchige Chanditha Hapuarachchi*, Carmen Koo, Relus Kek, Helen Xu, Yee Ling Lai, Lilac Liu, Suet Yheng Kok, Yuan Shi, Raphael Lee Tze Chuen, Kim-Sung Lee, Sebastian Maurer-Stroh, Lee Ching Ng

*Corresponding author

Supplementary Table S1. Summary of the number of samples screened and typed for DENV serotypes and the number of DENV-1 strains sequenced during the study period

**Period**	**Total No. of sera screened for dengue***	**No. of NS1 positive sera**	**No. of samples serotyped**		**No. of DENV-1 positives**	**No. of DENV-1 envelope genes sequenced**	**No. of envelope gene sequences of DENV-1 genotype III (epidemic lineage)**	**No. of DENV-1 genotype III (epidemic lineage) full genomes sequenced**
			**Real-time PCR**	**Conventional PCR**				
2012 (Nov-Dec)	271	26	18	6	8	8	8	1
2013 (Jan-Dec)	5742	1540	1243	178	820	728	626	50
2014 (Jan-May)	1931	618	531	50	517	452	424	32
Total	7944	2184	1792	234	1345	1188	1058	83

* Patient sera were screened by using the SD Bioline Dengue Duo kit (Standard Diagnostics INC., South Korea). The combo kit tests for the presence of both DENV NS1 antigen and IgM/IgG antibodies in a single assay. Only the sera received with patient consent are shown.

Supplementary Table S2. Amplification protocol and primers used for whole genome sequencing of DENV-1.

Fragment	Primer Name	Sequence (5' - 3')
Fragment 1	Den1-10FN*	TCTACGTGGACCGACAAGAAC
	Den1-552F	TGGATTTGGGAGAGTTWTGTGAG
	Den1-1102F	CGYAAACTGTGCATTGAAGC
	Den1-500R	ACTTTCCTCTTTCCTGCTTGC
	Den1-986R	CTCCTGACAGTCCTTCMACG
	Den1-1472R	GCTCCGTAGTCRGTCAGCT
	Den1-1949R*	CATGGTGCATCTGTTCCTTC
Fragment 2	Den1-1668F*	CGAAGAAGCAGGAAGTAGTCG
	Den1-2109F	TCAAGAAAGGAAGYAGCATAGG
	Den1-2869F	GAACATTTGGGAAGTTGAGGA
	Den1-2986R	CTTGATRGCAGCTGACATTAGC
	Den1-2509R	CTCTGTCCAAGTGTGRACTTCA
	Den1-3568R*	TTCTACTCCATCTGGAYCTCA
Fragment 3	Den1-3272F*	GTYGTTGTGGATGAACATTGTGG
	Den1-3710F	GCCACTTTYAGAATGAGACC
	Den1-4310F	TGGGAAGAAGAAGCAGAACAC
	Den1-4758F	ATATGGAGGAGGTTGGAGG

	Den1-4097R	GAAACATGGTTAGTGGTTTGC
	Den1-4587R	TAGATACCATCATCAAGAACTGC
	Den1-5166R*	CTCACGGACTATGGCTGGRAG
Fragment 4	Den1-4871F*	ACYCCTGAAGGCGAAGTTGG
	Den1-5361F	TGATYATCATGGATGAAGCACA
	Den1-5895F	GAGGAAGAATTGGAAGGAACCA
	Den1-6367F	AGGRAGAAGAAGTGTCTCAGGTGA
	Den1-6486R	CTTGTTCGGARTTGTGCAACA
	Den1-5928R	GTTCCTTCCAATTCTTCCTCTRCTC
	Den1-5550R	GAATGTCTCTTTCCTCATCTTGGAT
	Den1-6954R*	GCATAGAGRGTCCAGGCTGAAG
Fragment 5	Den1-6813FN*	TGACAGTRGCAGCCAATGAG
	Den1-7377F	CTCTTGATGCGGACYACATGG
	Den1-7820F	GTGGAAGAGGTGGCTGGTCA
	Den1-8441F	CAACATGGCATTATGATGAGGAC
	Den1-8851R	CACTGCTTCTTTTGCTGAGTTCC
	Den1-8261R	GACACAATGTTTCCTGTTCCACA
	Den1-7751R	GTTCCTCTCGACACTGCATGT
	Den1-7098R	CCTATGTCCATCTTCGATATTGGC
	Den1-9372R*	CCTCTYTGGTCACGTCTGGATA

Fragment 6	Den1-8984F*	GGRAGTCGTGCAATATGGTACA
	Den1-9352F	ATCCAGACGTGACCAGAGAG
	Den1-9754F	ATGCCGCAACCAAGATGAAC
	Den1-10217F	TACATGACATCAATGAAGAGATTC
	Den1-9418R	CATGTTGGTGAAAGTTTTAAGCC
	Den1-10031R	CCTCTATCCAAACCCTATTCCA
	Den1-10694R*	GTGCCTGGAATGATGCTGTAGA

Amplification primers are denoted by asterisks. The remaining primers, together with amplification primers were used for sequencing.

Amplification protocol

**PCR conditions - Fragments 1 - 2**		
**Temperature °C**	**Time**	**Cycle**
98 °C	10 sec	1
98 °C	5 sec	35
60 °C	10 sec	
72 °C	30 sec	
72 °C	2 min	1
20 °C	infinity	1

**PCR conditions - Fragments 3 - 6**		
**Temperature °C**	**Time**	**Cycle**
98 °C	10 sec	1
98 °C	5 sec	35
65 °C	10 sec	
72 °C	45 sec	
72 °C	2 min	1
20 °C	infinity	1

Each fragment was PCR amplified by using 0.5 µM of fragment-specific primers and 1X Phusion™ Flash High-Fidelity PCR Master Mix (Thermo Fisher Scientific, MA, USA).

Supplementary Table S3. Summary of the number of DENV-1 sequences used in each analysis

**Type of analysis**	**No. of study sequences used**	**Remarks**
**Based on envelope (*E*) gene sequences included in the study(n=744)**		
Definition of mutant spectra	744	The number of sequences of each variant mapped is indicated in Figure 1 legend
Calculation of p-distance	744	None
Median joining network analysis	715	Variant 13.03 (n=29) was not included in the analysis as the whole genome analysis revealed that variants 13.04-13.13 did not originate from 13.03
Spatial analysis using Geographical Information System	668	DENV-1 E gene sequences<br>Of 744 sequences included in the study, the location (postal code) information was available only for 668 sequences<br>The number of sequences of each variant mapped is indicated in Figure 4 legend
Calculation of mean substitution rate	291	*E* gene sequences were selected from 744 total sequences to represent each variant on a weekly basis from November 2012 to May 2014

Bayesian skyline plot analysis	291	*E* gene sequences were selected from 744 total sequences to represent each variant on a weekly basis from November 2012 to May 2014
**Based on whole polyprotein sequences included in the study(n=83)**		
Prediction of structural implications and immune interactions	83	None
Selection pressure analysis	83	Additional 81 DENV-1 whole polyprotein sequences retrieved from GenBank were also included in the analysis
Construction of maximum clade credibility tree	36	DENV-1 whole polyprotein sequences<br>The analysis also included 52 DENV-1 polyprotein sequences retrieved from the GenBank
Calculation of tMRCA	36	DENV-1 whole polyprotein sequences<br>The analysis also included 52 DENV-1 polyprotein sequences retrieved from the GenBank

Supplementary Video S1. Temporo-spatial distribution pattern of DENV-1 genotype III variants and total dengue cases. The video demonstrates the emergence and distribution of variants of the epidemic lineage on a weekly scale during the study period from November 2012 to May 2014. Each variant is colour-coded as shown in the figure legend. The coordinates of reported locations of 668 epidemic lineage strains were mapped and displayed in a video format

using R software package version 2.15.0 (http://www.R-project.org/) [59]. The distribution of total number of dengue cases has been shown in a density gradient (grey) as described in Figure 4.
